# 502. Early COVID-19 Treatment with SARS-CoV-2 Neutralizing Antibody Sotrovimab

**DOI:** 10.1093/ofid/ofab466.701

**Published:** 2021-12-04

**Authors:** Anil K Gupta, Yaneicy Gonzalez Rojas, Erick Juarez, Manuel Crespo Casal, Jaynier Moya, Diego Rodrigues Falci, Elias H Sarkis, Joel Solis, Hanzhe Zheng, Nicola Scott, Andrea L Cathcart, Christy Hebner, Jennifer Sager, Erik Mogalian, Daren Austin, Amanda Peppercorn, Elizabeth L Alexander, Wendy W Yeh, Almena Free, Cynthia Brinson, Melissa Aldinger, Adrienne Shapiro

**Affiliations:** 1 William Osler Health Centre, Kleinburg, Ontario, Canada; 2 Optimus U Corporation, Gonzalez MD and Aswad MD Health Services, Miami, Florida; 3 Florida International Medical Research, Miami, Florida; 4 Álvaro Cunqueiro Hospital, Pontevedra, Galicia, Spain; 5 Pines Care Research Center, Pembroke Pines, Florida; 6 Hospital de Clinicas de Porto Alegre, Porto Alegre, Rio Grande do Sul, Brazil; 7 Sarkis Clinical Trials, Gainesville, Florida; 8 Centex Studies, McAllen, Texas; 9 Vir Biotechnology, Inc., San Francisco, California; 10 GlaxoSmithKline, Stevenage, England, United Kingdom; 11 Vir Biotechnology, San Francisco, California; 12 Pinnacle Research Group, LLC, Anniston, Alabama; 13 Central Texas Clinical Research, Austin, Texas; 14 University of Washington and Fred Hutchinson Cancer Research Center, Seattle, Washington

## Abstract

**Background:**

COVID-19 disproportionately results in hospitalization and death in older patients and those with underlying comorbidities. Sotrovimab is a pan-sarbecovirus monoclonal antibody that binds a highly conserved epitope of the SARS-CoV-2 receptor binding domain and has an Fc modification that increases half-life. Sotrovimab retains activity against UK, S. Africa, Brazil, India, New York and California variants in vitro.

**Objectives:**

To evaluate the efficacy and safety of treatment with sotrovimab in high-risk, non-hospitalized patients with mild/moderate COVID-19, as part of the COMET-ICE clinical trial.

**Methods:**

Multicenter, double-blind, phase 3 trial in non-hospitalized patients with symptomatic COVID-19 and ≥1 risk factor for disease progression were randomized 1:1 to an IV infusion of sotrovimab 500 mg or placebo. The primary efficacy endpoint was the proportion of patients with COVID-19 progression, defined as hospitalization > 24 hours or death, due to any cause, ≤29 days of randomization.

**Results:**

The study met the pre-defined primary efficacy endpoint in a preplanned interim analysis: the risk of COVID-19 progression was significantly reduced by 85% (97.24% CI, 44% to 96%; P = 0.002) in 583 patients. In the final intention-to-treat analysis (N = 1057), the adjusted relative risk reduction was 79% (95% CI, 50% to 91%; p< 0.001) through Day 29 in recipients of sotrovimab (n=528) vs. placebo (n=529). Treatment with sotrovimab (ITT) resulted in a numerical reduction in the need for ER visits for illness management, hospitalization for acute illness management (any duration) or death (any cause) compared to placebo. No participants on sotrovimab required ICU admission, compared to 9 participants on placebo, of whom 4 participants required mechanical ventilation. No participants who received sotrovimab died, compared to 4 participants on placebo. The incidence of adverse events was similar between treatment arms and SAEs were numerically more common in the placebo arm.

**Conclusion:**

Treatment with sotrovimab 500 mg IV resulted in a clinically and statistically significant reduction in progression of COVID-19 to hospitalization or death in patients with mild/moderate disease and was well-tolerated.

**Study funding:**

GSK & VIR; NCT04545060

**Disclosures:**

**Jaynier Moya, MD**, **VIR Biotechnology** (Other Financial or Material Support, Jaynier Moya received non-financial support for serving as a clinical trial investigator for Vir Biotechnology) **Diego Rodrigues Falci, MD, MSc, PhD**, **Gilead Sciences** (Grant/Research Support, Scientific Research Study Investigator, Speaker's Bureau)**GSK** (Grant/Research Support, Scientific Research Study Investigator, Advisor or Review Panel member)**MSD** (Speaker's Bureau)**Pfizer** (Speaker's Bureau)**United Medical** (Speaker's Bureau, Other Financial or Material Support) **Joel Solis, MD**, **VIR Biotechnology** (Other Financial or Material Support, Joel Solis received non-financial support for serving as a clinical trial investigator for Vir Biotechnology) **Hanzhe Zheng, PhD**, **VIR Biotechnology** (Employee) **Nicola Scott, MSc**, **GlaxoSmithKline** (Employee, Shareholder) **Andrea L. Cathcart, PhD**, **Gilead** (Shareholder)**VIR** (Employee, Shareholder) **Christy Hebner, PhD**, **Vir Biotechnology** (Employee, Shareholder) **Jennifer Sager, PhD**, **GSK** (Other Financial or Material Support)**Vir Biotechnology** (Employee, Shareholder) **Erik Mogalian, PharmD, PhD**, **Vir Biotechnology** (Employee, Shareholder) **Daren Austin, PhD**, **GlaxoSmithKline** (Employee, Shareholder) **Amanda Peppercorn, MD**, **GlaxoSmithKline** (Employee) **Elizabeth L. Alexander, MD, MSc**, **GlaxoSmithKline** (Grant/Research Support, Other Financial or Material Support)**VIR Biotechnology** (Employee, Shareholder, GSK pharmaceuticals) **Wendy W. Yeh, MD**, **Vir Biotechnology** (Employee) **Almena Free, MD**, **Amgen** (Scientific Research Study Investigator)**Astra Zeneca** (Scientific Research Study Investigator)**Cardurian** (Scientific Research Study Investigator)**Coherus** (Scientific Research Study Investigator)**Freenome** (Scientific Research Study Investigator)**GlaxoSmithKline/Vir** (Scientific Research Study Investigator)**Ionis** (Scientific Research Study Investigator)**Kowa** (Scientific Research Study Investigator)**New Amsterdam** (Scientific Research Study Investigator)**Regenacy** (Scientific Research Study Investigator)**Romark** (Scientific Research Study Investigator)**Scynexis** (Scientific Research Study Investigator) **Cynthia Brinson, MD**, **Abbvie** (Scientific Research Study Investigator)**BI** (Scientific Research Study Investigator)**Gilead Sciences Inc.** (Scientific Research Study Investigator, Advisor or Review Panel member, Speaker's Bureau, Personal fees)**GSK** (Scientific Research Study Investigator)**Novo Nordisk** (Scientific Research Study Investigator)**ViiV Healthcare** (Scientific Research Study Investigator, Advisor or Review Panel member, Speaker's Bureau) **Melissa Aldinger, PharmD**, **VIR Biotechnology** (Employee) **Adrienne Shapiro, MD, PhD**, **Vir Biotechnology** (Scientific Research Study Investigator)

